# A Delicate Surgical Resection to Treat a Proximal Pulmonary Adenoid Cystic Carcinoma

**DOI:** 10.1155/2021/5529803

**Published:** 2021-07-02

**Authors:** N. Belloumi, H. Baili, M. Abdennadher, C. Habbouria, I. Bachouch, I. Bouassida, S. Zairi, F. Chermiti Ben Abdallah, S. Fenniche, H. Zribi, A. Marghli

**Affiliations:** ^1^Pulmonology Department Pavilion 4, Abderrahmen Mami Hospital, Tunisia; ^2^Faculty of Medicine of Tunis, University of Tunis El Manar, Tunisia; ^3^Thoracic Surgery Department, Abderrahmen Mami Hospital, Tunisia

## Abstract

Pulmonary adenoid cystic carcinoma (PACC) is an exceedingly rare tumor of low-grade malignancy. Diagnosis is often late, so the tumoral process may be huge at presentation. Surgical resection could be challenging, and the recurrence rate would be greater. We report, hereby, a case of proximal PACC with involvement of the carina in a young male adult, without respiratory distress. Surgical resection was performed through a left pneumonectomy followed by a complex trachea-bronchoplastic procedure. During the operative time, the assisted ventilatory mode was carefully chosen. No adjuvant treatment was needed. Our patient is still under clinicoradiological surveillance and remains disease-free.

## 1. Introduction

Pulmonary adenoid cystic carcinoma (PACC) is an exceedingly rare tumor of low-grade malignancy, accounting for 0.09 to 0.2% of all lung cancers [1]. Diagnosis is often late because the symptoms appear only after the tumor infiltrates the proximal airways. Symptoms are usually noncharacteristic, starting with cough and leading to dyspnea on exertion [2]. PACC has an unpredictable growth behavior. We report a case for broadening the sight of this disease, requesting a complex surgical procedure.

## 2. Case Report

**Figure 1:**
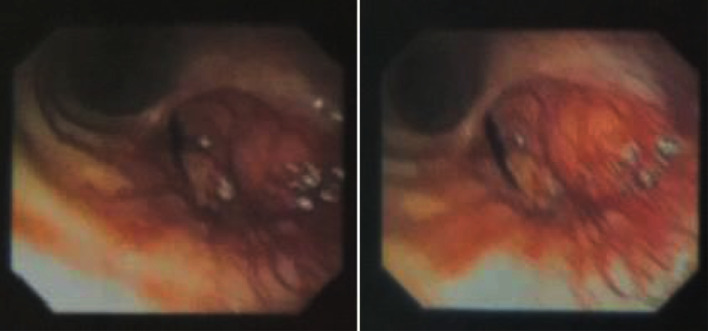
Bronchial endoscopy revealed a hypervascularized bud obstructing the left mainstem bronchus.

**Figure 2:**
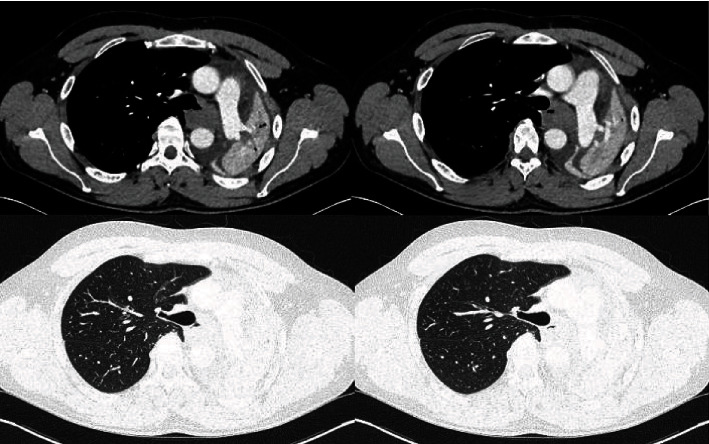
CT scan findings before the surgical procedure: a mediastinal gangliotumoral complex obstructing the left main bronchus, invasion of the carina and the lower trachea with an ipsilateral pulmonary collapse.

A 29-year-old male patient with no past medical history was admitted to our department to explore a right basal thoracic pain mentioned two months ago aggravated by an acute onset dyspnea. He was an active smoker and worked as a baker. On examination, the patient was afebrile and eupneic, no oxygen was needed, and auscultation found a decrease in vesicular murmur on the left with dullness on percussion. Chest X-ray revealed a homogeneous pulmonary opacity occupying the entire left pulmonary field with a mediastinum lifted to the left. Bronchial endoscopy revealed a hypervascularized bud obstructing the entrance to the left mainstem bronchus (Figure 1). The anatomopathological exam of bronchial biopsies showed a bronchial mucosa infiltrated by a carcinomatous proliferation arranged in tubular structures, cribriform masses, small lobules, and cords. Tumor cells were monomorphic, cuboidal, or basophilic with sparse cytoplasm and little atypical dense chromatin ovoid nuclei. Mitoses were rare. The stroma was fibrohyaline with myxoϊd islands and several layers of mucoid material. This histological appearance was very suggestive of the diagnosis of adenoid cystic carcinoma. CT scan had shown a mediastinal gangliotumoral complex of 52 × 38 mm obstructing the left main bronchus with an ipsilateral pulmonary collapse. The process invaded the carina and the lower trachea (Figure 2). No distant metastasis was found.

The patient underwent a left pneumonectomy with a section of the left mainstem bronchus and lymph node dissection through a left thoracotomy. Then, a right thoracotomy was performed. Selective high-frequency jet ventilation was performed through the right main bronchus. The trachea-bronchial section was enlarged to the lower part (4 cm) of the trachea and the carina. A terminoterminal anastomosis restored continuity of the distal part of the trachea and the right mainstem bronchus. Anastomotic sutures using 4-0 polydioxanone suturing thread were placed around the entire circumference, then ligated making ends meet.

**Figure 3:**
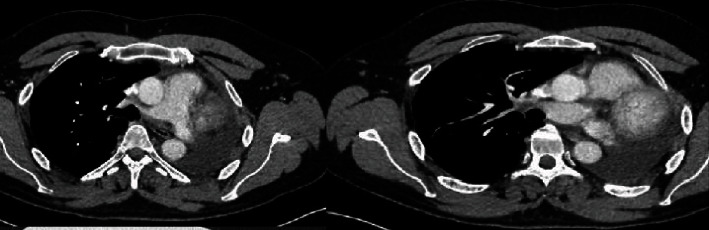
CT scan after left pneumonectomy.

Postoperatively, the patient was given oxygen therapy at home for a month. The CT scan performed 9 months after surgery showed no signs of regional or distant recurrence. Endoscopic control objectified a barely visible bulge at the right strain bronchus that marks trachea-bronchial plasty without narrowing of the lumen. A clinicoradiological follow-up is ensured for 24 months nowadays (Figure 3). The patient regained his professional career.

## 3. Discussion

PACC is uncommon but represents the predominant type of salivary gland-type lung carcinomas [3]. So far, several large studies and more case reports have been reported in the literature about PACC. This tumor is unrelated to smoking, it appears usually in the fifth decade, and there is no particular sex predilection [2, 4, 5]. Our patient was, so, younger than usually reported.

ACC develops mostly in the trachea, the mainstem, or the lobar bronchi [6, 7]. The tumor is presumed to derive from a primitive tracheobronchial gland cell. PACC is usually solitary, nodular, or polypoid masses that protrude the cartilage and infiltrate the peribronchial tissue [6, 7].

Histologically, ACC shows differentiation towards ductal and myoepithelial cells [6]. Authors already described cribriform, tubular, or solid patterns. The most important and classical feature is the “cribriform” pattern where nests of tumor cells have a soft yellow-white cut surface. Most ACC show a pattern mixture. Immunostaining is not necessary for the diagnosis but can serve out in several special cases notably in solid or tubular ACCs [2]. Tumor cells express smooth muscle actin and myosin, the protein S100, and CD117 [8]. Overexpression of p53 and Ki-67 with loss of myoepithelial markers was found in high-grade tumors.

ACC tends to submucosal extension linked to the arrangement of the accessory salivary glands, located between the cartilage and the tracheal fibromuscular membrane, at the level of the posterior wall of the trachea. It shows up in CT scan as an intraluminal mass extending through the tracheal wall. Sometimes, the lesion may present as a circumferential parietal thickening [2, 9]. Respiratory functional assessment is mandatory after the diagnosis of ACC. The fixed airway obstruction with decreased inspiratory and peak expiratory flow is due to the intraluminal narrowing [1].

Treatment options for advanced PACC patients are limited. Currently, the best treatment relies on complete resection with tracheobronchial plasty depending on tumoral location and extension. Developed surgical and anesthesiological procedures would make even wide tumors resectable. Contraindications to surgery include mediastinal lymph nodes, vascular involvement, tracheal invasion for more than 50% of its length, and distant metastases [10].

The extent of surgical resection and adjuvant radiotherapy may influence the prognosis of ACC. In a 30-year study of ACC of the airway, Calzada et al. found a high progression-free survival rate for patients who underwent surgical resection followed by adjuvant radiotherapy [11]. Early radical resection and radiotherapy are associated with a low risk of local recurrence of PACC.

## 4. Conclusions

Lung adenoid cystic carcinoma is a rare lung cancer with protracted but unpredictable growth behavior. Diagnosis and treatment are not yet fully consensual. For huge resectable masses, postoperative radiation would cure the microscopically residual tumor of the mediastinum. The mainstay of treatment is based upon combined surgical resection to radiotherapy. A long-term follow-up is required due to the risk of local or distant recurrence.

## Data Availability

Data are available upon request to the corresponding author, email = nidhalbelloumi@gmail.com.
